# Bipolar Disorder and Bone Mineral Density Z-Scores in Relation to Clinical Characteristics and Lithium Medication

**DOI:** 10.3390/jcm11237158

**Published:** 2022-12-01

**Authors:** Anders Lassas, Karl-fredrik Norrback, Rolf Adolfsson, Martin Maripuu

**Affiliations:** Department of Clinical Sciences, Pychiatry, Umeå University, 901 87 Umeå, Sweden

**Keywords:** bipolar disorder, osteoporosis, lithium, disease burden

## Abstract

Bipolar disorder is associated with a long range of medical comorbidities, including migraine, diabetes, and cardiovascular disease. Bipolar disorder has also been associated with an increased risk of bone fractures. Osteoporosis is a reduction in bone mineral density, which leads to an increased risk for fragility fractures. Currently there is limited research on the association between bipolar disorder and osteoporosis. We aimed to study the association between high and low bone mineral density in relation to disease and treatment history in a sample of bipolar patients. We found that bipolar patients with high bone mineral density were more often on lithium medication, had a more active lifestyle and expressed lower current disease burden. Low mineral density was not associated with any of the addressed aspects of disease and treatment history. In conclusion our results support that patients on lithium treatment have higher bone mineral density; further studies are needed to address if lithium medication causes an increase in bone mineral density, and lowers the risk of bone fractures in bipolar disorder.

## 1. Introduction

Bipolar disorder (BD) is a chronic disorder characterized by states of depression and hypomania/mania. With a prevalence of 1–2% of the world’s population, it is one of the leading causes of disability in young people [1]. Suicide and medical conditions such as cardiovascular disease are the leading causes of death, and persons with BD tend to die almost 10 years earlier, compared to the general population. BD has also been linked to a range of both psychological and medical comorbidities, including attention deficit hyperactivity disorder, substance-related disorders, anxiety disorder, obsessive-compulsive disorder, migraine, diabetes, and cardiovascular disease [2,3]. BD has also been associated with an increased risk of bone fractures [4,5,6] and osteoporosis [7].

Osteoporosis is estimated to affect 200 million people worldwide and is defined by a reduction in bone mineral density (BMD), which leads to an increased risk for fragility fractures. Fragility fractures are associated with a significant decline in the quality of life and a high degree of morbidity and mortality [8]. BD is genetically related to schizophrenia (9), and the recurrent depressive episodes of BD and recurrent episodes of unipolar depression are similar. Unipolar depression and schizophrenia have both been associated with osteoporosis and reduced BMD [9,10]. The association between BD and osteoporosis have been less clear [11], but recent studies conclude that patients with BD compared to the general population have a higher risk of fractures and osteoporosis [4,5,6,7,12]. However, the risk of low BMD seems to be lower in bipolar depression than in unipolar depression and schizophrenia [13].

BD is associated with chronic physiological stress, and patients have been shown to be symptomatic about 50% of the time, which leads to a high accumulative stress load over time [14]. In line with this, disturbances in the hypothalamic-pituitary-adrenal axis have repeatedly been identified in patients with BD [15]. Traditionally BD has mostly been seen as a cognitive disease; however, recent studies indicate that inflammation plays a role in BD pathophysiology both in the brain and in the periphery [16,17,18]. Chronic stress, glucocorticoids, and inflammation are all risk factors for secondary osteoporosis [19,20]. Unhealthy lifestyle choices, such as smoking and alcohol use, are also more prevalent in patients with BD and constitute risk factors for secondary osteoporosis [11].

Lithium is the first line of treatment in BD, and lithium treatment is often life-long and aims to prevent depressive and manic episodes. The exact mechanism of action for mood stabilization is not known, but multiple mechanisms are involved [21]. One suggested pathway of action is the inhibition of GSK3β, which leads to the activation of the Wnt signaling pathway, which is associated with a range of processes, one of them being bone formation [22]. Lithium has been associated with higher BMD and a decreased risk of fractures [23,24,25,26], and animal studies have also shown that lithium enhances bone formation in mice [27]. A recent large Danish register study also supported that lithium medication in bipolar disorder was associated with decreased risk of osteoporosis [7]. A positive effect of lithium on BMD might have masked osteoporosis in some earlier studies of osteoporosis in BD.

To summarize, bipolar disorder seems to be associated with osteoporosis and increased risk of fractures. Lithium medication tends to decrease these risks. However, there is sparse knowledge about which disease factors and which treatment strategies in bipolar disorder are associated with higher or lower BMD. Therefore, we aimed to study the association between high and low BMD in relation to disease and treatment history in a sample of bipolar patients.

## 2. Materials and Methods

### 2.1. Study Participants

The study included 149 patients with BD I or II, as defined by DSM-IV. The study material was collected between 1998 and 2007 at the affective unit at the Psychiatric Clinic at the University Hospital in Umeå, Sweden. A somatic examination along with semi-structured psychiatric interviews were performed. The semi-structured interviews included FIGS (Family Interview for Genetic Studies, https://www.nimhgenetics.org/interviews (accessed on 15 June 2022), the MINI International Neuropsychiatric Interview [28], and the Beck Depression Inventory [29]. Physical activity was self-estimated. Medical history in the form of electroconvulsive therapy, suicide attempts, and medication were collected retrospectively from patients’ medical records. Excluded from the study were patients with current mania or hypomania, neurological disorders affecting the central nervous system, schizoaffective disorder, related patients, current use of oral corticosteroids, and any other condition that could lead to problems completing the study protocol. Eight patients were receiving estrogen substitution therapy, which is often used as a prophylactic treatment against osteoporosis after menopause [30]. Three patients used either Eulexin (flutamid), Testogel (testosterone gel), or Tamoxifen (tamoxifen), all of which potentially can affect BMD. Etalpha (alfacalcidol) is a drug for disturbances in calcium metabolism and was used by one patient, but the patient’s diagnosis was unknown. One patient suffered from Morbus Crohn, which can affect BMD. The present study was part of a series of studies using partly the same material; for further information see [15].

### 2.2. Dual-Energy X-ray Absorptiometry Scans

BMD was measured with dual-energy X-ray absorptiometry scans.

### 2.3. Z-Score

Primary osteoporosis is defined as osteoporosis caused by age [19]. Because we were interested in secondary causes for low BMD, we used the Z-score to quantify the standard deviation of BMD after adjusting for age, and sex [19]. Thus, a Z-score of zero is the average BMD for a group of patients with the same age, and sex. We analyzed the average Z-score for different measurement sites. In order to observe any bone-strengthening effects of lithium, the average Z-score was further analyzed in different patient subgroups with and without lithium medication. Lithium prophylaxis longer than 1 year was chosen as the cut off because we hypothesized that any potential effect of lithium on BMD would only be seen after a considerable length of lithium use.

We were interested in clinical characteristics from the patients’ medical histories in relation to Z-scores. Thus, a range of clinical characteristics were obtained from medical records and personal interviews. The characteristics were compared to the L2–L4 Z-score because secondary osteoporosis often affects the spinal column first [31]. We further decided to divide the patients into low (Z-score ≤ −1), middle (−1 < Z-score < 1), and high (Z-score ≥ 1) L2–L4 groups for comparison between groups. We chose these cutoffs to compare individuals within the first standard deviation to individuals with relatively high or relatively low BMD in relation to age and sex. The low group included 33 (22.1%) patients, the middle group 78 (52.3%) patients, and the high group 38 (25.5%) patients. In a final logistic regression, clinical characteristics that might influence the Z-score were included. The choice of parameters depended both on results from our current study and on previous studies. We included the following characteristics: age of onset, disease duration, lithium prophylaxis > 1 year and the quality of life. The model was additionally adjusted for sex, age, and the type of BD.

### 2.4. Statistical Methods

The IBM SPSS version 25.0.0.1 (https://www.ibm.com/support/pages/downloading-ibm-spss-statistics-25) (accessed on 1 June 2022) was used for data analysis. Single-sample *t*-tests were used to calculate the average Z-scores for different dual-energy X-ray absorptiometry measurement locations, and Mann-Whitney U-test were used to calculate the differences in clinical characteristics between Z-score groups. Fischer’s exact test was used for categorical data, and a binary logistic regression model was also performed. A *p*-value below 0.05 was considered significant.

## 3. Results

### 3.1. Descriptive

The study included 149 patients, of which 59 were male and 90 were female, with a mean age of 46.3 years and a range of 19–79 years. Of the patients, 37.6% were diagnosed with BD II and 62.4% with BD I, with an average disease duration of 24.3 years. Lithium was the most-used current medication with a prevalence of 52.3%. The average BMI of the group was 26.2 kg/m^2^. For further statistics, see Table 1.

### 3.2. Z-Scores in Bipolar Disorder

The mean Z-scores from different measurement sites are shown in Table 2. The whole group’s average femur Z-score (0.276, *p* = 0.001) was significantly higher than the age, sex adjusted average of zero. The L1 Z-score (−0.236, *p* = 0.015) was significantly lower than zero, while the L2–L4 Z-score did not differ significantly. Patients with lithium prophylaxis < 1 year had a similar distribution with a significantly higher femur Z-score, significantly lower L1 Z-score, and a L2–L4 Z-score that did not differ significantly. Patients with lithium prophylaxis > 1 year had significantly increased L2–L4 and femur Z-scores and a significantly decreased L1 Z-score.

### 3.3. Clinical Characteristics and Z-Scores

Clinical characteristics in relation to Z-score are shown in Table 3. No significant differences were observed in the low compared to the middle and high L2–L4 Z-score groups, while there were several clinical characteristics associated with a high L2–L4 Z-score compared to middle and low L2–L4 Z-score. Patients in the high versus middle and low group were more often on current lithium medication, they had been on lithium medication for a longer time and had more often been on lithium prophylaxis > 1 year. A significantly higher portion of patients in the high Z-score group had an active lifestyle, they also scored lower on the BDI scale, and higher on the QOL Scale. The previous year’s Global Assessment of Functioning score was also higher in the high Z-score group, while there was no difference in the current score. Further, the current use of antiepileptics was significantly lower in the high Z-score group. Known risk factors for low BMD such as smoking, and BMI did not differ significantly between the groups.

A binary logistical model comparing different Z-score groups is shown in Table 4. No significant differences were seen when comparing the low Z-score group versus the middle and high Z-score groups. When comparing the high versus middle and low groups, high WHOQOL (OR= 1.142, *p* = 0.029) and lithium prophylaxis > 1 year (OR = 3.246, *p* = 0.026) was significantly associated with increased Z-score.

## 4. Discussion

Bipolar patients with high BMD were more often on lithium medication, had a more active lifestyle, and expressed lower current disease burden in the aspect of higher quality of life, higher global functioning, and a lower degree of depressive symptoms.

Patients with a high Z-score had a lower current disease burden, scoring lower on depression scales and having a higher quality of life. A lower disease burden could indicate a less chronic burden of BD or an overall lower stress burden, which might be a reason for the higher Z-score. A lower disease burden might also influence lifestyle choices, such as choosing a more active lifestyle. We had expected that clinical characteristics from the medical history that indicated more severe or chronic disease would be more prevalent in patients with low BMD. However, no clinical characteristics of low Z-scores were observed. This was surprising to us and we have no clear-cut explanation for this. It is possible that some factors, such as low physical activity or medication with antiepileptics or SGA, could have turned significant in a larger study sample. However, previous studies on antipsychotics or antiepileptics and fracture risk have not found any significant associations [5,23], and low BMD have been shown to be more common in bipolar disorder already at the time of diagnosis [12]. It is possible that some of the reasons for low BMD and the increased risk of fractures in bipolar disorder should be searched for earlier in bipolar patients’ lives. However, the increased rate of fracture has been associated with a higher rate of psychiatric hospitalization in patients with BD, although this was not supported in our analysis of BMD [5]. Social factors and lifestyle choices, other than a more active lifestyle, did not differ across different Z-scores.

Lithium has been associated with increased BMD, osteoporosis, and lower fracture risk [7,23,24]. Interestingly, patients with a high Z-score used lithium more frequently and more often had used lithium > 1 year. In the adjusted logistical regression, there was still a higher odds ratio (OR = 3.2) for the patients with lithium prophylaxis > 1 year to belong to the high Z-score group. The average femur and L2–L4 Z-scores were higher for patients with lithium > 1 year. The L2–L4 Z-score was not significantly different for the whole group or for patients with lithium < 1 year. All this suggests that lithium has a positive effect on BMD. However, this study is cross sectional and cannot address if lithium cause higher BMD.

A high Z-score was associated with a lower use of antiepileptics, but this could possibly be a consequence of the lower use of lithium prophylaxis in this subgroup. Another unexpected result was that patients with a longer disease duration had higher Z-scores, and this might be explained by longer time on lithium and is supported by the fact that the result was not significant in the logistic model in which lithium medication was included.

Our study population did not have decreased Z-scores (i.e., lower BMD than an international control group). Only the L1 Z-score was significantly lower, while the femur Z-score was significantly higher and the L2–L4 Z-score did not differ significantly from the average population. When taking lithium into account, and looking at the average score for patients with lithium prophylaxis less than 1 year, the distribution of the results was the same. The results are somewhat inconclusive in that the average Z-score depended on the measurement site. However, variations in Z-score depending on the measurement site are common [26]. According to previous studies, other chronic mental illnesses, such as unipolar depression and schizophrenia, are associated with low BMD [8,9]. Although little is known about BMD in BD, our results are not in line with previous studies that have shown that patients with BD had a higher occurrence of fractures [4,5] and osteoporosis [7]. However, those studies did not look at the relationship between BD and BMD, and the risk of fractures could be associated with other aspects of a bipolar life. This study relied on an international control sample to calculate the Z-score, and it is possible that the use of a local control sample might have yielded different results. This has been observed in both a Swedish and a Norwegian study, where the use of local control samples increased the degree of osteoporosis in the study group compared to results based on the international Z-score [32,33].

The most important limitations of the study are the cross-sectional design, the lack of a local control group, and that physical activity was self-assessed. Several analyses were based on the L2–L4 Z-score, because this is a region with early bone loss in secondary osteoporosis. However, L2–L4 analysis might give falsely elevated values in the elderly because of vertebral osteophytosis, vertebral end plate and facet sclerosis, osteochondrosis, or aortic calcification [34]. On the other hand, there were no age differences between Z-score groups. We did not include analyzes of thyroid dysfunction, hormone system dysregulation, diabetes, rheumatologic, or autoimmune diseases, which all could affect BMD; neither did we exclude patients on medication that could affect BMD. This choice was made based on the fact that we did not know the history of medication except for medication with mood stabilizers. The strengths of the study are the relatively large study population and the use of detailed medical histories, especially the detailed history of lithium use.

In conclusion, high BMD within the bipolar group was associated with lithium medication and an active lifestyle, and lower current disease burden. We found no general association between low BMD and BD. This should, however, be interpreted with caution since results are based on the international control sample and studies in Norway and Sweden indicate higher normal BMD in our populations. Further studies, preferably with local control samples, are needed to replicate these results.

## Figures and Tables

**Table 1:** Demographics.

Clinical Characteristics	
Age, years (IQR)	48.0 (21.0)
Disease duration, years (IQR)	24.3 (12.8)
Age at onset, years (IQR)	18.0 (12.0)
Female Sex, %	60.4
Bipolar type II, %	37.6
First-degree heredity for bipolar disorder, %	47.7
Suicide attempt, %	38.3
Passed menopause, % of women	44.3
**Treatment history**	
Treatment delay, years (IQR)	11.0 (15.7)
Disease duration without lithium prophylaxis, years (IQR)	19.4 (18.4)
Disease duration without “any mood stabilizer”, years (IQR)	17.4 (18.2)
Lithium prophylaxis, years (IQR)	1.5 (7.9)
“Any mood stabilizer”, years (IQR)	2.2 (8.4)
Lithium prophylaxis > 1 year, %	59.7
Hospitalization, %	72.5
ECT, %	24.2
**Current medication**	
Lithium, %	52.3
Antiepileptics, %	26.2
SGA, %	12.8
No current mood stabilizer, %	17.4
FGA, %	11.4
Antidepressives, %	27.5
Sedative/anxiolytic, %	35.6
**Lifestyle/Social demographics**	
Schooling, years (IQR)	12.0 (3.0)
Smoker, %	22.8
BMI, kg/m^2^ (IQR)	25.5 (5.1)
Inactive lifestyle, %	14.1
Very active lifestyle, %	14.8
**Self-rated current disease burden**	
BDI, (IQR)	7 (17)
BAI, (IQR)	7 (10)
WHOQOL, (IQR)	14.0 (5.5)
GAF (current), (IQR)	80 (25)
GAF (last year), (IQR)	70 (30)

IQR, Inter Quartile Range; ECT, electroconvulsive therapy; BMI, body mass index; FGA, first-generation antipsychotics; SGA, second-generation antipsychotics; BDI, Beck Depression Inventory; BAI, Beck Anxiety Inventory; WHOQOL, The World Health Organization Quality of Life; GAF, Global Assessment of Functioning.

**Table 2:** Z-scores and bipolar disorder.

				95% Confidence Interval	
	Measurement Location	Mean (SD)	*p*-Value	Lower	Upper
**Whole group**	Z-score femur	0.276 (1.03)	0.001	0.109	0.443
	Z-score L2–L4	0.164 (1.34)	0.137	−0.053	0.382
	Z-score L1	−0.236 (1.17)	0.015	−0.425	−0.047
**Lithium prophylaxis < 1 year**	Z-score femur	0.297 (1.10)	0.041	0.013	0.580
	Z-score L2–L4	−0.063 (1.34)	0.716	−0.410	0.284
	Z-score L1	−0.373 (1.16)	0.016	−0.673	−0.073
**Lithium prophylaxis > 1 year**	Z-score femur	0.262 (0.99)	0.014	0.053	0.470
	Z-score L2–L4	0.318 (1.33)	0.026	0.039	0.597
	Z-score L1	−0.143 (1.17)	0.252	−0.389	0.103

One-sample *t*-test was used to calculate the means. L2–L4, lumbar vertebrae 2–4; L1, lumbar vertebrae 1.

**Table 3:** Z-score in relation to clinical characteristics (Median/IQR).

KERRYPNX	Z-Score L2–L4			Low vs. Mid Z-Score	High vs. Mid Z-Score
	Low (*n* = 33)	Mid (*n* = 78)	High (*n* = 38)	*p*-Value	*p*-Value
**Clinical characteristics**					
Age, years (IQR)	48.0 (17.0)	46.5 (24.0)	51.0 (24.0)	0.969	0.078
Disease duration, years (IQR)	28.0 (21.0)	25.0 (20.3)	29.5 (23.5)	0.602	0.098
Age of onset, years (IQR)	18.0 (12.0)	19.5 (13.0)	18.5 (11.0)	0.379	0.981
Frist-episode depression, %	87.9	82.1	86.8	0.785	0.797
Female sex, %	48.5	65.4	60.5	0.157	1.000
Bipolar type II, %	33.3	38.5	39.5	0.685	0.847
First-degree heredity for bipolar disorder, %	54.5	50.0	36.8	0.431	0.136
Suicide attempt, %	42.4	37.2	36.8	0.685	1.000
Passed menopause, % of women	56.3	40.8	43.5	0.405	1.000
**Treatment history**					
Treatment delay, years (IQR)	14.0 (24.5)	13.0 (18.3)	15.5 (18.3)	0.821	0.721
Disease duration without lithium, years (IQR)	21.9 (25.5)	18.3 (17.2)	20.7 (20.2)	0.412	0.543
Disease duration without any mood stabilizer, years (IQR)	17.9 (26.0)	16.3 (17.4)	20.2 (19.6)	0.452	0.470
Lithium prophylaxis, years (IQR)	1.3 (5.2)	1.0 (5.1)	2.4 (10.7)	0.655	0.004
Any mood stabilizer, years (IQR)	1.9 (8.6)	1.8 (8.1)	4.5 (12.1)	0.456	0.011
Lithium prophylaxis > 1 year, %	54.5	52.6	78.9	0.548	0.007
Hospitalization, %	75.8	69.2	76.3	0.825	0.675
ECT, %	27.3	16.7	36.8	0.649	0.047
**Current medication**					
Lithium, %	42.4	47.4	71.1	0.237	0.009
Antiepileptics, %	27.3	33.3	10.5	1.000	0.010
SGA, %	18.2	11.5	10.5	0.373	0.782
No current mood stabilizer, %	24.2	17.9	10.5	0.298	0.225
FGA, %	15.2	14.1	2.6	0.534	0.072
Antidepressives, %	27.3	25.6	31.6	1.000	0.533
Sedative/anxiolytic, %	33.3	33.3	42.1	0.839	0.334
**Life style/Social demographics**					
Schooling, years (IQR)	12.0 (3.0)	12.0 (3.0)	12.0 (5.8)	0.927	0.740
Smoker, %	24.2	23.1	21.1	0.817	0.827
BMI, kg/m^2^ (IQR)	25.6 (4.7)	26.4 (5.5)	24.7 (5.8)	0.817	0.124
Inactive/sedentary lifestyle, %	18.2	15.6	7.9	0.571	0.282
Very active lifestyle, %	12.1	9.1	28.9	0.425	0.008
**Self-rated current disease burden**					
BDI (IQR)	7.0 (17.0)	10.0 (21.0)	4.0 (10.0)	0.724	0.012
BAI (IQR)	5.5 (8.8)	9.0 (11.5)	5.5 (11.0)	0.216	0.333
WHOQOL (IQR)	14.0 (6.0)	13.0 (7.0)	15.0 (6.0)	0.705	0.024
GAF (current) (IQR)	85.0 (21.0)	79.0 (30.0)	85.0 (50.0)	0.293	0.179
GAF (last year) (IQR)	70.0 (27.5)	70.0 (28.5)	80.0 (24.3)	0.941	0.042
Overall health	3.0 (1.0)	4.0 (1.25)	4.0 (1.25)	0.705	0.028

All values are medians. Mann-Whitney U-test was used to compare groups. For categorical data, Fischer’s exact test was used. ECT, electroconvulsive therapy; BMI, body mass index; FGA, first-generation antipsychotics; SGA, Second-generation antipsychotics; BDI, Beck Depression Inventory; BAI, Beck Anxiety Inventory; IQR, Inter Quartile Range; WHOQOL, The World Health Organization Quality of Life; GAF, Global Assessment of Functioning; L2–L4, lumbar vertebrae 2–4; L1, lumbar vertebrae 1.

**Table 4:** Z-scores in relation to clinical characteristics, logistic regression.

Clinical Characteristics	High L2–L4 Z-Score vs. Middle and Low L2–L4 Z-Score		Low L2–L4 Z-Score vs. Middle and High L2–L4 Z-Score	
	Odds Ratio	*p*-Value	Odds Ratio	*p*-Value
Age of onset	0.977	0.347	0.995	0.842
Disease duration	1.010	0.604	1.015	0.438
Female Sex	0.869	0.739	0.513	0.104
Bipolar type II	1.957	0.130	0.672	0.388
Lithium prophylaxis > 1 year	3.246	0.026	0.593	0.287
WHOQOL	1.142	0.029	0.985	0.794

A binary logistic model was used to compare groups. WHOQOL, The World Health Organization Quality of Life; L2–L4, lumbar vertebrae 2–4; L1, lumbar vertebrae 1.

## Data Availability

The data presented in this study are available on request from the corresponding author. The data are not publicly available due to ethical and privacy reasons.
